# Interferometry with non-classical motional states of a Bose–Einstein condensate

**DOI:** 10.1038/ncomms5009

**Published:** 2014-05-30

**Authors:** S. van Frank, A. Negretti, T. Berrada, R. Bücker, S. Montangero, J.-F. Schaff, T. Schumm, T. Calarco, J. Schmiedmayer

**Affiliations:** 1 Vienna Center for Quantum Science and Technology, Atominstitut, TU Wien, Stadionallee 2, A-1020 Vienna, Austria; 2 Zentrum für Optische Quantentechnologien, Universität Hamburg, The Hamburg Centre for Ultrafast Imaging, Luruper Chaussee 149, D-22761 Hamburg, Germany; 3 Institut für Quanteninformationsverarbeitung, Universität Ulm, Albert-Einstein-Allee 11, D-89069 Ulm, Germany; 4 Max Planck Institute for the Structure and Dynamics of Matter, Bldg. 99 (CFEL), Luruper Chaussee 149, D-22761 Hamburg, Germany; 5 Center for Integrated Quantum Science and Technology (IQST), Universität of Ulm, Albert-Einstein-Allee 11, D-89069 Ulm, Germany

## Abstract

The Ramsey interferometer is a prime example of precise control at the quantum level. It is usually implemented using internal states of atoms, molecules or ions, for which powerful manipulation procedures are now available. Whether it is possible to control external degrees of freedom of more complex, interacting many-body systems at this level remained an open question. Here we demonstrate a two-pulse Ramsey-type interferometer for non-classical motional states of a Bose–Einstein condensate in an anharmonic trap. The control sequences used to manipulate the condensate wavefunction are obtained from optimal control theory and are directly optimized to maximize the interferometric contrast. They permit a fast manipulation of the atomic ensemble compared to the intrinsic decay processes and many-body dephasing effects. This allows us to reach an interferometric contrast of 92% in the experimental implementation.

## Abstract (web summary)

Ramsey interferometers are used to measure minute energy shifts, but they are usually only applied to simple, non-interacting ensembles. Here, the authors demonstrate a two-pulse Ramsey-type interferometer built on the motional states of an interacting Bose–Einstein condensate using optimal control.

## Graphical abstract

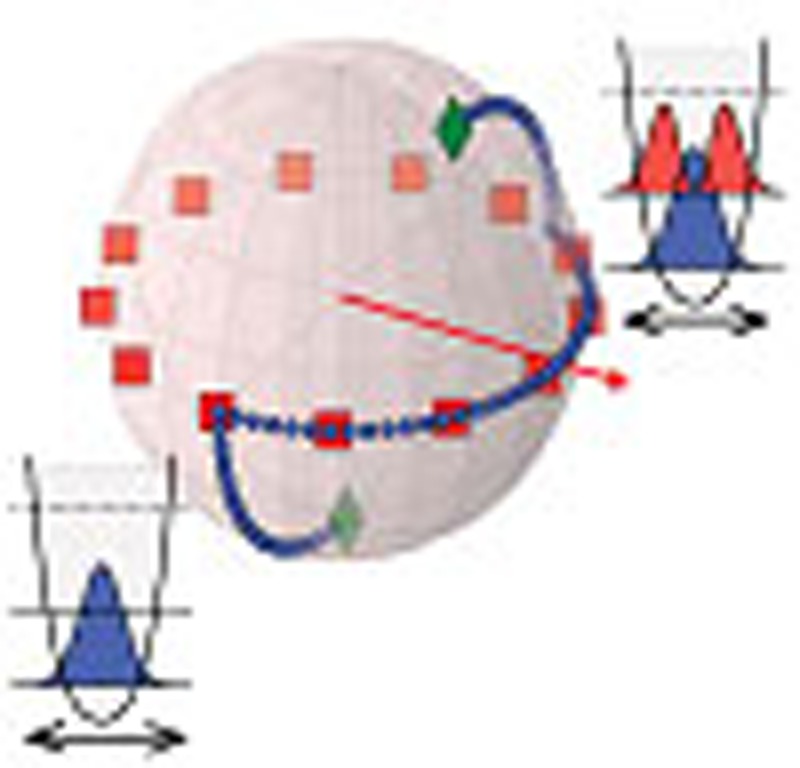

Fundamental investigations and technological applications of quantum physics are rapidly expanding research fields^1^. Essential elements for their development are the progress made in the control of quantum states and the improvement of powerful techniques like spectroscopy and interferometry. A prominent example is the method of separated oscillating fields^2^,^3^, as it combines accurate quantum control with interferometry.

This technique, refered to as Ramsey interferometry, has become has become an essential tool to investigate the physics of well-isolated, single-particle quantum systems or non-interacting ensembles. Its applications range from the measurement of nuclear magnetic moments, for which it was originally conceived, to molecular spectroscopy^4^, and from atomic clocks^5^ to cavity quantum electrodynamics experiments^6^.

Implementing Ramsey interferometry for many-body systems is challenging. Interactions between the constituents lead to complex dynamics, which require new approaches to implement the ‘Ramsey pulses’—namely, the two successive oscillatory fields realizing ‘*π*/2’ rotations in Ramsey’s original work. A key obstacle here, compared to single-particle or non-interacting systems, is the lack of separation between the different energy scales.

Realizing a Ramsey interferometer for the motional states of a Bose–Einstein condensate (BEC) in a trap requires the following operations: (1) the creation of an equal superposition of two trap eigenstates with a controlled relative phase; and (2) a pulse acting as a phase-sensitive *π*/2 operation for all these superpositions for the read-out (Fig. 1c). These operations must be fast and preserve phase coherence over the entire BEC.

An excited BEC exhibits complex behaviour, in particular in the presence of intrinsic dephasing^7^,^8^, decoherence or decay^9^, which also make coherent manipulation challenging^10^. One strategy to control the quantum states of such systems is to implement operations faster than the characteristic timescales of the prejudicial processes, using optimal control theory (OCT)^11^,^12^. The speedup can be exploited to realize elaborate manipulations, as in the present case of a sequence of transfer pulses for interferometry.

We drive transitions between motional states by displacing (‘shaking’) the trap along one axis (Fig. 1a), following a trajectory obtained by OCT. By making the trapping potential anharmonic with a strong quartic component along the shaking direction^13^, we effectively reduce the external states of the BEC to a two-level system. Unlike in the harmonic case, the resonant frequencies between each pair of states are then different, enabling the design of OCT pulses that suppress leakage to higher motional states. This ‘shaking’ method was first introduced in ref. 9 to realize a full population inversion of the two lowest-lying motional states and study the subsequent decay dynamics^14^.

We design the two control pulses using the chopped random basis algorithm (CRAB)^15^, with the particularity that the second pulse is directly optimized to reach high interferometric contrast. For this optimization, we describe the system’s dynamics as a condensate wavefunction using an effective one-dimensional Gross-Pitaevskii equation (1D GPE), which is justified by the very low temperatures and the very short times considered. The OCT pulses allow us to drive transitions between motional states^13^ on a timescale comparable to the trapping frequency^16^. The produced states are then superpositions of two motional Fock states^17^,^18^, to be distinguished from the Poissonian superpositions of motional states populated in a classical centre-of-mass movement.

In this work, we demonstrate phase-sensitive coherent control of the motional states of a many-body system by realizing, using optimal control, a Ramsey-type interferometer with a contrast of 92% experimentally. This application to Ramsey interferometry proves that manipulating a complex, interacting many-body system in a fast, coherent and reproducible way is possible.

## Results

### Experimental procedure

**Figure 1:**
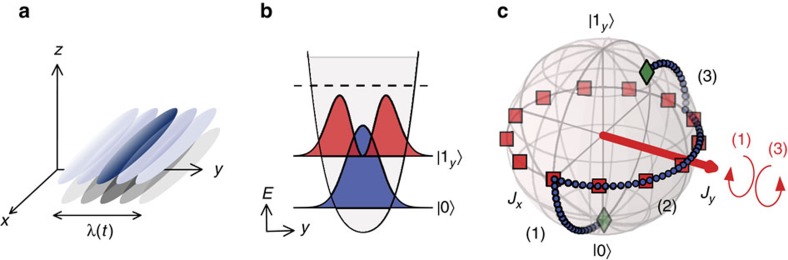
Schematic of the Ramsey interferometric sequence. (**a**) Representation of the BEC subjected to a fast displacement *λ*(*t*) in the *y*-direction. (**b**) Trapping potential and effective two-mode system. The anharmonicity in the *y*-direction leads to a unique transition frequency between the ground state |0› (blue) and the lowest-lying excited state |1_*y*_› (red), effectively almost isolating the two-level system |0›−|1_*y*_›. The other states (dashed line) have higher energies. (**c**) Example of an interferometric trajectory (blue dots) on the Bloch sphere representation of the two-level system. (1) is the first pulse that prepares a balanced coherent superposition. (2) is the phase accumulation time corresponding to a rotation around the vertical axis. (3) is the second pulse, which is equivalent to a *π*/2 pulse for the states on the equator and corresponds to a 90° counter-clockwise rotation around *J*_*y*_. The red squares show the 15 points on which the second pulse was optimized.

Our experimental system, sketched in Fig. 1, is a dilute, quasi one-dimensional quantum-degenerate gas of ~700 ^87^Rb atoms in an elongated magnetic trap on an atom chip^19^. Both the temperature (*T*<50 nK≈*h*/*k*_B_ × 1 kHz) and chemical potential (*μ*/*h*≃0.6 kHz) (*k*_B_ being Boltzmann’s constant and *h* Planck’s constant) are below the smallest transverse level spacing (*E*_01_=*h* × 1.83 kHz), ensuring that the system is initialized in its motional ground state |0› (see Methods). The trapping potential is made anisotropic and anharmonic in the horizontal transverse *y*-direction by radio-frequency dressing^13^,^20^,^21^. It is well approximated by the 6th-order polynomial


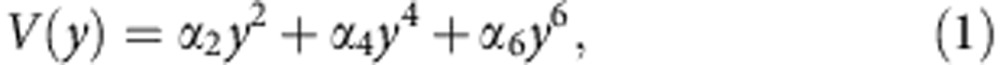


with 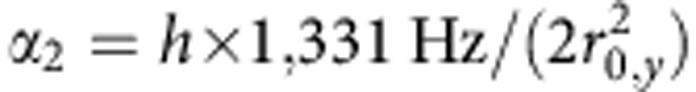, 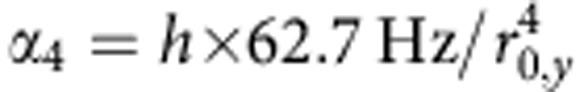 and 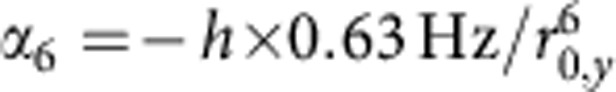, where *r*_0,*y*_=252 nm is the r.m.s. radius of the single-particle ground-state wavefunction in the *y*-direction. The energy differences between the three lowest transverse single-particle levels of the potential are *E*_01_=*h* × 1.83 kHz and *E*_12_=*h* × 1.98 kHz. In the other directions, the confinement remains essentially harmonic with *ω*_*z*_=2π × 2.58 kHz and *ω*_*x*_=2π × 16 Hz.

**Figure 2:**
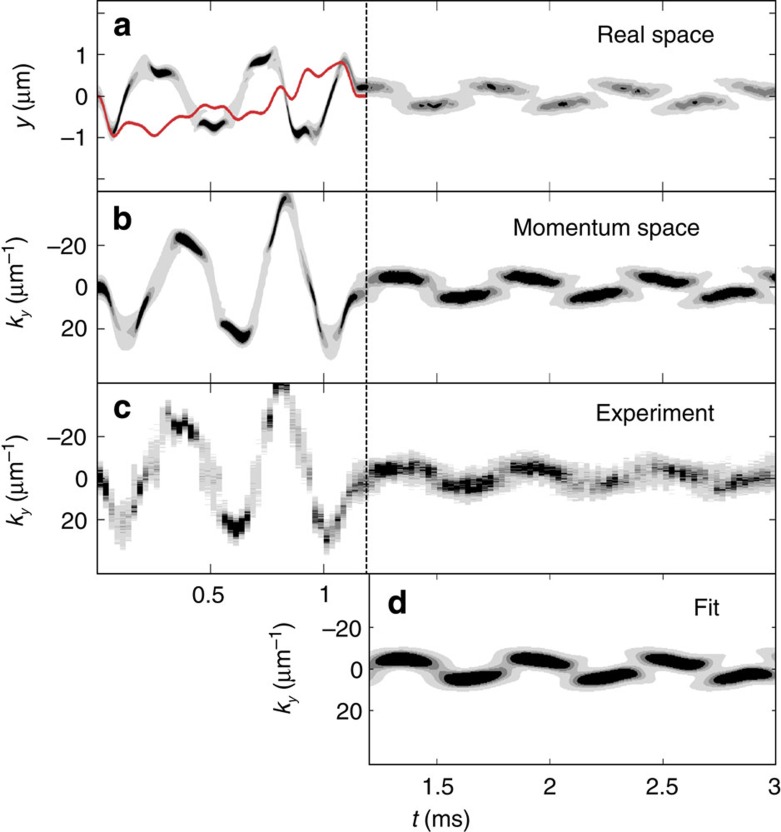
Dynamics of the excitation and interference patterns observed during and after the first pulse. (**a**) *In situ* transverse (along *y*-direction) density profile as a function of time during and after the first pulse. Red line: real space trajectory of the excitation pulse *λ*(*t*). The displacement of the trap minimum corresponds to several times the ground state r.m.s. size. (**b**) Simulated picture of the momentum distribution during and after the first pulse. (**c**) Measured momentum distribution during and after the first pulse. The time-of-flight images were averaged over three repetitions, integrated along the longitudinal *x*-direction and concatenated to show the time evolution. (**d**) Fit to the momentum distribution from which the populations *p*_0_ and *p*_1_ are extracted (see text).

We drive transitions between the two lowest-lying motional states by shaking the trap purely along the *y*-direction, following trajectories obtained by the CRAB optimization. The trap displacement *λ*(*t*) reaches values on the order of 4 times the r.m.s. size of the ground-state wavefunction (see Fig. 2a).

### Optimization with the CRAB algorithm

The goal of optimal control is to find the best path in the control parameter space, which is expressed formally as a minimization of a cost function or performance measure^22^,^23^. For the optimization of the pulses, we describe the system as a condensate wavefunction using an effective one-dimensional GPE along the *y*-axis, with the Hamiltonian


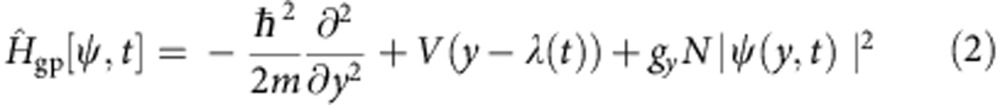


where *ℏ* is the reduced Planck constant, *m* the atomic mass, *N* the number of atoms and *g*_*y*_ the effective one-dimensional interaction constant in the *y*-direction^24^. The minimum of the potential *V* can be spatially displaced along *y* by a distance *λ*(*t*) (see Fig. 1a).

The CRAB optimization method expands the control pulse into a (not necessarily orthogonal) basis. Here, the optimization is carried out on 60 Fourier components with their respective amplitudes and phases. Under the action of the control pulse, the wavefunction undergoes a transformation that is computed numerically using the split-step analysis method^25^. The wavefunctions of the different motional states are the stationary solutions of the GPE and were obtained numerically by imaginary time propagation^26^.

### State analysis

The behaviour of the wavefunction in the horizontal *xy*-plane at different times *t* throughout the Ramsey sequence is monitored by time-of-flight fluorescence imaging^27^. Along the transverse *y*-axis, the high trap frequency and 46ms expansion time ensure that the measured atomic density is an image of the in-trap momentum distribution. The experimental images are integrated along the longitudinal *x*-axis and concatenated to follow the evolution of the transverse wavefunction over time, as illustrated in Fig. 2c.

After the control pulses, the density distributions exhibit characteristic ‘beating’ patterns arising from interferences between the different motional levels populated. To simulate this distribution, we calculate the evolution of the 1D GPE in the static potential *V*(*y*) starting from a given initial superposition of *k* states:


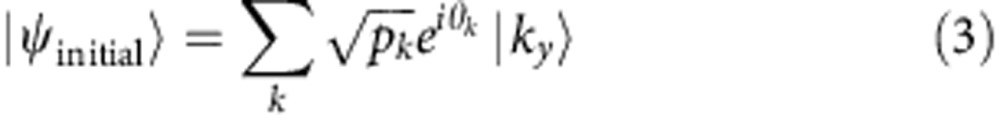


where *k*ε{0,1,2}, corresponding to the three lowest-lying states in the *y*-direction. We compute the momentum distribution and compare its evolution to the experimental densities after time-of-flight. A fitting procedure enables us to infer which superposition of motional states is most likely to have generated the experimentally observed beating patterns, in particular what the populations of interest for the interferometer, *p*_0_ and *p*_1_, are. This way, we can estimate the fidelity of the first pulse as well as the output of the full interferometric sequence (see Methods and ref. 13).

### First pulse

The first pulse (Fig. 2a) aims to create a balanced superposition 
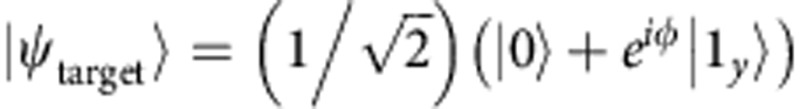
 of the ground state |0› and first excited state |1_*y*_› with a relative phase *φ* arbitrarily chosen to be zero. The cost function to be minimized can be written in terms of the overlap fidelity :


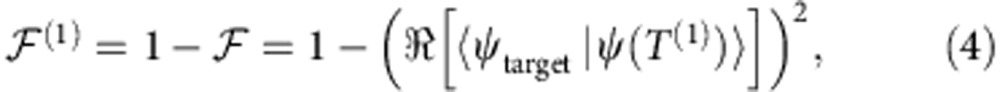


where |*ψ*(*T*^(1)^)› represents the state of the system at the end of the first pulse.

When designing the pulse, a trade-off must be found between fidelity and speed^16^,^28^. We choose a pulse with a theoretical fidelity of 99% for a pulse duration of 1.19 ms. This duration is about twice the timescale set by the single-particle level spacing 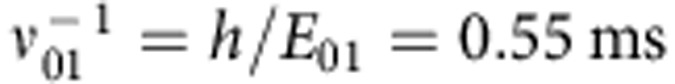. The experimental realization yields an overlap of 95(4)% of the obtained wavefunction with |*ψ*_target_› (see Methods).

### Phase accumulation time

**Figure 3:**
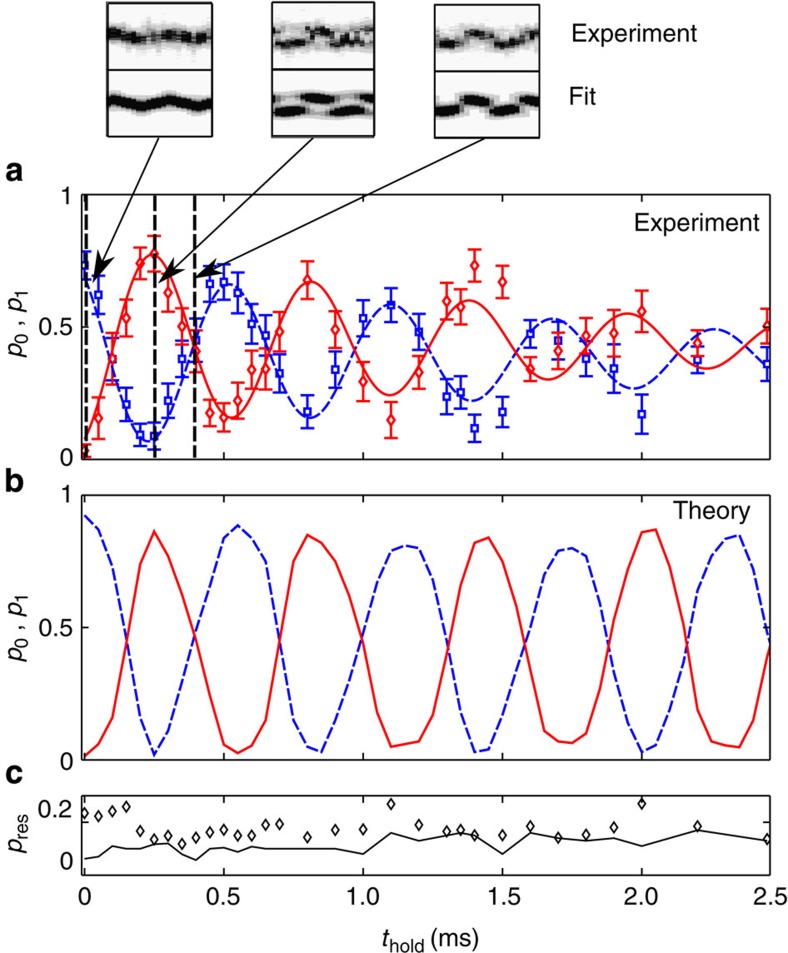
Interference fringes of the motional-states interferometer. (**a**) Experimental data. Populations of the ground state *p*_0_ (blue squares) and first excited state *p*_1_ (red diamonds), extracted from fits to the experimental density patterns, as a function of the phase accumulation time *t*_hold_. The error bars indicate the 1*σ* confidence interval of the fit. The blue and red dashed lines are exponentially damped sines. (**b**) OCT optimization data. Populations of the ground state *t*_hold_ (blue dashed line) and first excited state *p*_1_ (red line) as a function of the phase accumulation time *t*_hold_. (**c**) Populations in higher excited states in the optimization (black solid line) compared to residual part in the fits to experimental data (black diamonds). The top insets are examples of experimental momentum distributions (upper) and their corresponding fitted GPE momentum distribution (lower) for the three different hold times indicated by the vertical dashed lines in panel (**a**).

After creating a coherent superposition of |0› and |1_*y*_›, the wavefunction is held in a static potential for an adjustable time *t*_hold_. The energy difference between the levels leads to an evolution of the relative phase. In a simplified linear picture, this phase evolution corresponds to a rotation of the state vector on the equatorial plane of the Bloch sphere at a constant angular frequency given by the energy difference between the levels (see Fig. 1c). In the trap, the inter-atomic interactions introduce a non-linearity in the system and the corresponding mean-field energy slightly decreases the frequency with respect to the single-particle energy splitting^13^ *E*_01_. For a balanced superposition, one period of the oscillation of the relative phase is then *T*=0.58 ms, corresponding to a 5% increase with respect to the single-particle precession period. The phase accumulation time *t*_hold_ is varied to observe interferometric fringes in the Ramsey sequence (Fig. 3).

### Full Ramsey sequence

The second pulse is also implemented by shaking the trapping potential in the *y*-direction. However, contrary to the first pulse, it does not target a specific state superposition starting from a known initial state. It rather aims at transforming any state on the equator of the Bloch sphere into another state superposition, where the populations of |0› and |1_*y*_› are maximally sensitive to the phase of the initial state. To optimize this pulse, the following cost function was minimized:


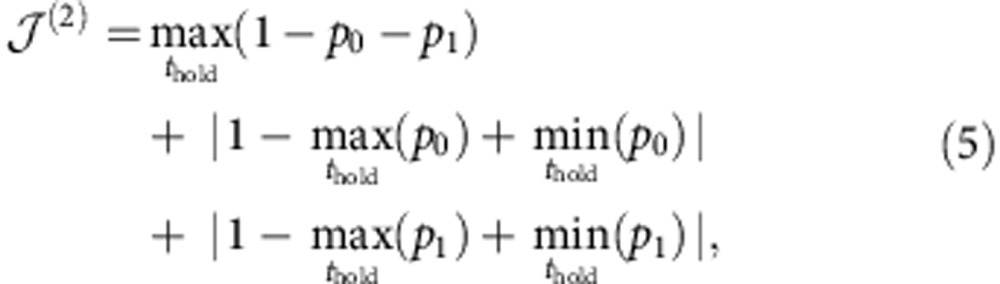


where *p*_0_ (respectively *p*_1_) is the ground state (respectively first excited state) population at the end of the second pulse, and the maximum is taken over *N*_*h*_=15 different values of the phase accumulation time *t*_hold_ for which the numerical optimization was performed. The first term of equation (5) minimizes the transfer of population to higher-energy levels, while the second term (respectively third term) maximizes the amplitude of the oscillation of *p*_0_ (respectively *p*_1_). The obtained pulse has a duration of 1.6 ms. It can also be seen as a *π*/2 pulse, or 90° rotation around the *J*_*y*_-axis, for the states on the equator as depicted in Fig. 1c.

We point out that this optimization procedure aims at maximizing the visibility, and not directly at producing a *π*/2 pulse. In the latter case, the optimization can be carried out using a different cost function, for example =1−min_*φ*_(|‹*ψ*_0_(*φ*)|*ψ*(*φ*)›|^2^), where *φ* is an angle in the equatorial plane of the Bloch sphere, *ψ*_0_(*φ*) is the state obtained when applying a real *π*/2 pulse to an initial state described by *φ*, and *ψ*(*φ*) is the actual state produced by the control sequence when applied to the same initial state. Using this alternative approach leads to nearly as good results in terms of visibility.

When simulating the whole interferometric sequence, we observe an oscillation of *p*_0_ and *p*_1_ as a function of *t*_hold_, with a periodicity of 0.58 ms. The contrast, defined as (*p*_*i*_)=(max(*p*_*i*_)−min(*p*_*i*_))/(max(*p*_*i*_)+min(*p*_*i*_)), reaches (*p*_0_)≈(*p*_1_)≈97% in the numerical simulations. As shown in Fig. 3c, a limited transfer of population to higher excited states of the order 10% also takes place. We note that although the second pulse is designed without constraint on the shape of the interferometric fringes, the final fringe evolution is close to a sine function.

Experimentally, the populations *p*_0_ and *p*_1_ for different phase accumulation times are inferred from the evolution of the momentum density after the two-pulse Ramsey sequence, like the one represented in Fig. 2c, for these different phase accumulation times. The populations of the superpositions are extracted using our state analysis (see Methods for details). Fig. 3a shows the obtained Ramsey signal. The experimental results are in good agreement with the numerical simulation on the first interferometric fringes. The contrast reaches 92(5)%, and the Ramsey period measured is 0.57(2) ms. The fit residuals, interpreted as a population in higher excited states and an incoherent fraction, amount to 15%–25% depending on *t*_hold_.

## Discussion

The goal of the cost function we chose to optimize the second pulse is to maximize the visibility of the interferometer fringes, rather than generating a general *π*/2 pulse. This pulse was optimized for a finite number of points on the equator of the Bloch sphere. However, we point out that the holding times *t*_hold_ (and with it the phases in the superposition) chosen for the experiment differ from the ones used for the numerical optimization of the second pulse. The experimental observation of fringes indicates that the pulse is valid for all points on the equator.

Looking at longer times *t*_hold_ we observe a reduction of contrast, indicating a loss of coherence in the created superposition over time. Fitting an exponentially damped sine to the experimental fringes reveals a damping time constant of 1.6±0.7 ms. This decay is not observed in our 1D GPE simulation (see Fig. 3b).

We investigated four possible mechanisms that could explain the contrast reduction. However, none of them could account for the observed decay. First, perturbations of the wavefunction could arise from a coupling between the different transverse and longitudinal modes. However, simulations using a 3D GPE solver revealed no such effect. Second, we evaluated the rate of dephasing^29^,^30^ between the two modes arising from interactions and binomial number fluctuations in each mode and found *R*~52 mrad ms^−1^, hence a dephasing of 1 rad only after ~20 ms, which is too long to account for the observed decay (see Methods for details). Third, the phase fluctuations present in a 1D geometry^31^ do not affect us directly, as the system is mono-mode along the *y*-direction, where we drive and observe the dynamics. But they could potentially affect the read-out contrast; however, these are not observed on our experimental timescale. Finally, collisional decay of the quantum gas trapped in the first excited state, like in ref. 9, would lead to emission of momentum-correlated atom pairs. We do not observe such pair creation in the present experiment, although this can also be due to the lower population in |1_*y*_› and the shorter observation times compared to refs 9, 14.

Other types of collisions could take place between atoms in the same quantum state, between atoms in the ground and first excited states, or with residual atoms in highly excited states. A detailed calculation of the decoherence effects listed here would require tools that are only partially available in the state-of-the-art numerical simulations of such systems, and in any case is beyond the scope of this paper.

In conclusion, we have demonstrated a scheme to coherently control non-classical motional states with high speed and efficiency using optimal control, and implemented it in a two-pulse Ramsey interferometer sequence, realizing experimentally a motional state interferometer with a contrast higher than 90%.

This proves that coherent manipulation of a complex, interacting many-body system in a reproducible way is possible on timescales shorter than the natural timescale given by the energy differences of the internal many-body states. Similar procedures will be relevant for a large class of schemes in the context of quantum information and quantum metrology. In addition, the ability to precisely prepare complex, highly excited states makes our approach a valuable tool for the study of many-body dynamics.

Generally, Ramsey interferometry using motional states introduces a new tool to study out-of-equilibrium evolution of coherent systems at the quantum level^32^. This may help to shed light on the mechanisms responsible for the loss of coherence in many-body systems, and in particular show the role of interactions. As a specific example, our system can be viewed as a leaking qubit exhibiting mean-field coupling and decoherence, and could be used as a quantum simulator for solid-state qubits^33^.

In addition, fast and coherent manipulation of motional states in a many-body quantum system offers many possibilities that go far beyond Ramsey interferometry. It permits the implementation of general gate operations, the encoding of information into motional states^34^ and, more generally, opens up new perspectives for the use of many-body systems as a viable element in quantum technological applications.

## Methods

### Trapping potential and displacement

The one-dimensional trapping potential is realized on an atom chip^35^ by a radially symmetric Ioffe–Pritchard field modified by radio-frequency dressing^20^,^36^, as explained in detail in ref. 13. In the present experiment, the AC current applied has a peak-to-peak amplitude *I*_RF_=20 mA with detuning *δ*=−54 kHz with respect to the Larmor frequency near the trap minimum (*ν*_0_=824 kHz).

In the *y*-direction, where the shaking occurs, the potential is well approximated by the 6th-order polynomial given in the main text. In the *z*-direction, it can be described by a quartic polynomial of the form 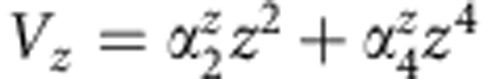, with the coefficients 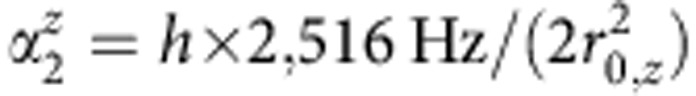 and 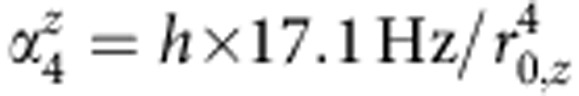, where *r*_0,*z*_=212 nm is the oscillator length in this direction. This gives a first-level spacing 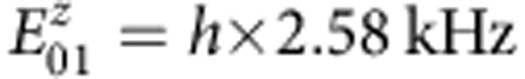.

To create motional states superpositions, we shake the trap minimum along the *y*-direction. This displacement is achieved by modulating the radio-frequency currents with a low-frequency signal. The frequencies of this signal, on the order of a few kilohertz, are much lower than the Larmor frequency of the atoms but higher than the limit for adiabatic displacement of the wavefunction in the transverse potential. This modulation displaces the potential minimum along *y*, following a control trajectory calculated by OCT. The atomic cloud is shaken by this fast potential displacement.

The effect of interactions is to shift the levels slightly, which requires to take them into account in the optimization of the ramp.

### First pulse cost function

The fidelity of the first pulse is expressed as (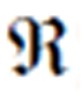
[‹*ψ*_target_|*ψ*(*T*^(1)^)›])^2^ instead of the more general |‹*ψ*_target_|*ψ*(*T*^(1)^)›|^2^. The motivation to use the real part is related to the fact that we assumed the GPE eigenstates |*ψ*_0_›, |*ψ*_1_› to be real-valued functions, and therefore |*ψ*_target_› is a real function while |*ψ*(*T*^(1)^)› is a complex function. Then, the relevant part of the scalar product is only its real part, as shown below.

We took as goal state 
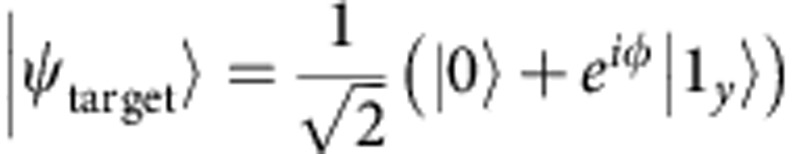
.

If the final state at the time *T* after the application of the first pulse is


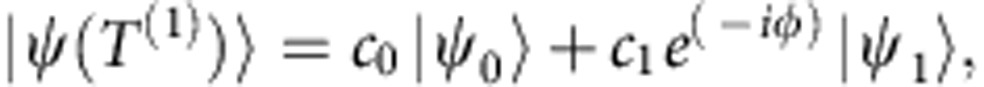


with *c*_0_ and *c*_1_ being real numbers, then the square of the scalar product is


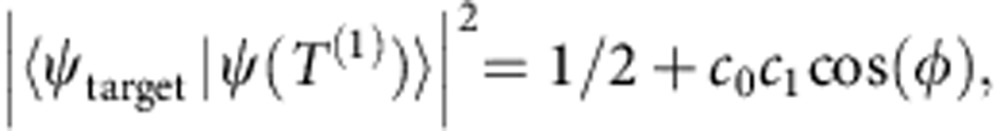


while the real part squared is


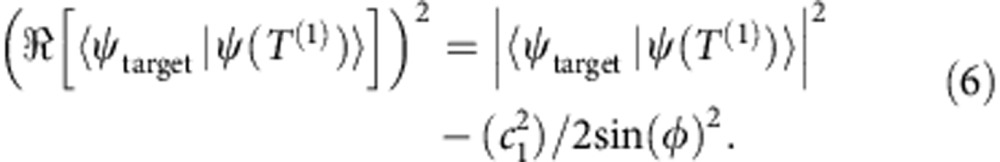


The desired state corresponds to *φ*=2*πn* with *n*ε*Z*; both definitions yield the same results.

### Fitting procedure and error estimation

To recover the wavefunction superposition after the control pulse from experimental data, we fit the data with a time-dependent momentum density along *y*. The numerical momentum density is obtained by Fourier transform of an in-trap GPE simulation. Experimentally, we can access the atomic density after 46 ms time-of-flight. The fast transverse expansion of the cloud due to high confinement causes the atomic interactions to become rapidly negligible, hence the expansion can be considered ballistic. In the limit of infinite expansion time, the in-trap momentum distribution and the density after time of flight are strictly equivalent. Here, the time of flight is sufficiently long to make this assumption. If we express the momenta as wave numbers *k*_*y*_, a distance δ*y* in the experimental image then corresponds to δ*k*_*y*_=*α*δ*y*, with *α*=*m*/*ħt*_TOF_≈0.03 μm^−2^. The simulated momentum distribution is slightly rescaled on the *k*-axis and corrected for imaging broadening, then sampled to match the experimental sampling time, *t*=0.05 ms.

We fit the time-dependent momentum-space density with that obtained from the state 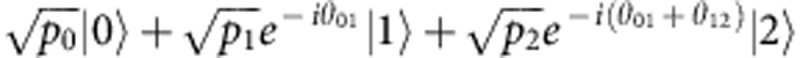, where *p*_0_, *p*_1_, *p*_2_, *θ*_01_ and *θ*_12_ are fit parameters. We chose to restrict the model to a three-states superposition here. First, multi-mode simulations show that the main features of the experimental data can be reproduced by a three-mode description similar to ref. 30. Second, this assumption is justified by the fact that adding more states does not improve nor modify much the output of the fit.

To obtain the combination of parameters most likely to have generated the observed momentum distribution density, we use a simplex regression method that searches the smallest possible residual and gives their corresponding best values for the fit parameters. Once these parameters are obtained, we look for the uncertainty of the fit by estimating the variances and co-variances of the different parameters and deduce the confidence intervals of the fit.

We note that these fits are based on Gross–Pitaevskii simulations, which represent a unitary evolution for a mean-field description of a system at zero temperature. Although this model describes the main features of our data very well, some discrepancy between the model and the experiment (for example, many-body or finite temperature effects) may have systematic effects on the estimation of the fidelity. It is nevertheless unlikely that these discrepancies have a qualitative effect on the interferometer output.

### Phase diffusion

We estimated the rate of many-body dephasing that could arise from number fluctuations in the ground and excited states. We followed the approach of ref. 30, and, assuming weak interaction, approximated the field operator 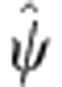, describing the BEC by


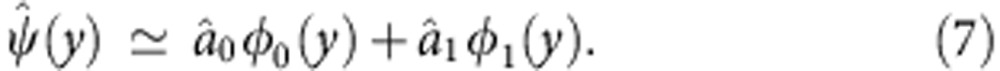


Here the *φ*_*i*_ are the two lower-lying eigenstates of the non-interacting part of the Hamiltonian (taken to be real and normalized to ∫|*φ*_*i*_|^2^d*y*=1), the 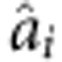 are annihilation operators associated with the modes and a 1D geometry along the *y*-axis was assumed for simplicity. From the full many-body Hamiltonian describing the condensate and equation (7), we obtain the following effective two-mode Hamiltonian:


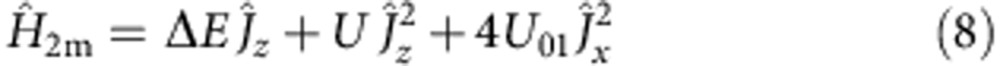


with


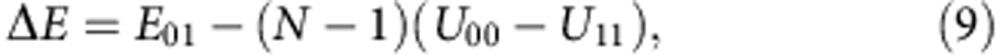



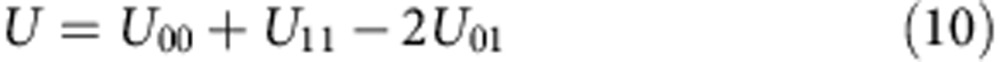


and


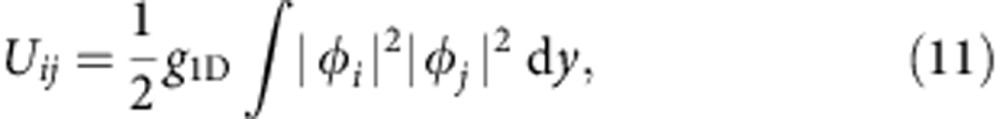


where we used the usual spin representation for the many-body two-level system by introducing the operators 
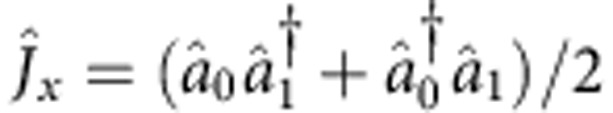
, 
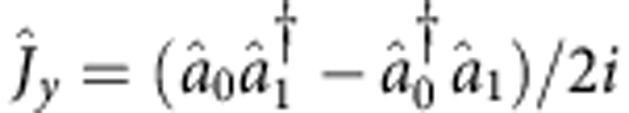
 and 
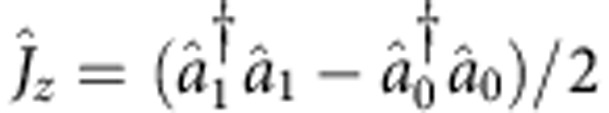
, which satisfy angular momentum commutation relations. This Hamiltonian resembles the Bosonic Josephson Hamiltonian in the presence of an energy offset between the two modes, here given by the difference of chemical potential between the ground and first excited states (first term 
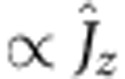
). The second term 
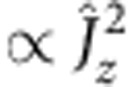
, which comes from interactions, is responsible for ‘phase diffusion’ (dephasing). It leads to squeezing at short times^37^, generation of strongly non-classical states^38^ and a loss of coherence at longer times^29^,^39^. The third term is generally neglected in bosonic Josephson junctions due to the weak overlap between the modes.

In the second term of equation (8), it is apparent that phase diffusion is reduced compared to, for example, the case of a double-well system^8^, as the modes have a significant spatial overlap. This is similar to the case of a spinor condensate in which two spin states share the same external wavefunction and have similar scattering lengths^40^,^41^. We can evaluate the phase diffusion rate if we assume, for example, a binomial distribution of the atoms in each mode (that is, 
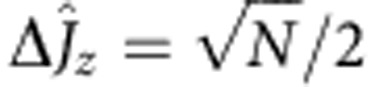
), which is a fair assumption if the first pulse is performed quickly compared to the other energy scales (in particular compared to interactions that may induce squeezing). It is then given by^29^,^39^


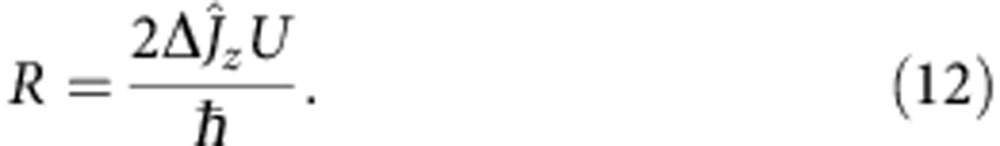


We computed the two wavefunctions *φ*_0_ and *φ*_1_ in the trapping potential *V*_*y*_ and obtained the energies *U*_00_/*h*=0.34 Hz, *U*_11_/*h*=0.26 Hz and *U*_01_/*h*=0.15 Hz. This yields *U*/*h*=0.31 Hz, and a phase diffusion rate *R*=52 mrad ms^−1^. This rate increases with atom number fluctuations and can become significant if the fluctuations are much stronger than in the binomial case (
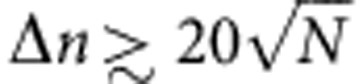
, *n* being the population difference between the modes). This could be the case, as both modes overlap.

## Author contributions

T.S. and J.S. conceived the experiment. J.S. and T.C. led the scientific questions. S.v.F. performed the experiments. S.v.F., R.B. and J.-F.S. analysed the data. A.N. and S.M. carried out the numerical simulations and optimizations. All the authors contributed to the elaboration of the project and helped in editing the manuscript.

## Additional information

**How to cite this article:** van Frank, S. *et al.* Interferometry with non-classical motional states of a Bose–Einstein condensate. *Nat. Commun.* 5:4009 doi: 10.1038/ncomms5009 (2014).
